# Generation of 3D Hemogenic Gastruloids From Mouse Embryonic Stem Cells

**DOI:** 10.21769/BioProtoc.5777

**Published:** 2026-08-05

**Authors:** Ylenia Cicirò, Cristina Pina, Denise Ragusa

**Affiliations:** 1 Sanquin Research, Landsteiner Laboratory, Amsterdam, The Netherlands; 2 Department of Biosciences, College of Health, Medicine and Life Sciences, Brunel University of London, Uxbridge, United Kingdom; 3 Centre for Genome Engineering and Maintenance, College of Health, Medicine and Life Sciences, Brunel University of London, Uxbridge, United Kingdom; 4 Cancer Center Amsterdam, Amsterdam UMC, Amsterdam, The Netherlands; 5 Princess Máxima Centre for Pediatric Oncology, Utrecht, The Netherlands

**Keywords:** Gastruloid, Development, Hemogenic endothelium, Hematopoiesis, Embryonic stem cell differentiation 3D model

## Abstract

Embryonic blood formation encompasses the independent generation of different cell types in distinct cellular and anatomical environments, reflecting highly coordinated specific hierarchies of interacting tissues. Despite widespread use of embryonic stem cells (ESC) and induced pluripotent stem cell (iPSC)-based models to attempt to capture blood development in vitro and generate hematopoietic stem cells (HSC), a system that fully captures the spatial and temporal complexity of embryonic hematopoiesis is still lacking. In recent years, gastruloid models have emerged as powerful representations of early development, demonstrating self-organizing behaviors such as symmetry breaking, elongation, multi-axis formation, somitogenesis, and early organogenesis, with striking parallels to embryonic processes. Here, we present a protocol to generate hemogenic gastruloids (haemGx) from mouse ESC (mESC) that closely recapitulates the multi-stage, multi-niche process of blood formation and generates developmentally accurate hematopoietic progenitors. The haemGx model has been proven valuable in understanding embryonic hematopoiesis, as well as an in vitro model of forms of infant leukemia with an embryonic, in utero origin.

Key features

• The haemGx protocol allows the generation of developmentally accurate endothelial and hematopoietic precursor and progenitor cell types.

• The haemGx protocol achieves a level of spatiotemporal control that closely recapitulates key aspects of embryonic development.

• The haemGx protocol is compatible with multiple mESC lines, enabling reproducible generation of developmental blood cell types across different genetic backgrounds.

• At endpoint, individual haemGx reaches ~800–1,000 μm in diameter and contains 30,000/50,000 cells. The 96-well format allows for upscaling and high-throughput applications.

## Graphical overview


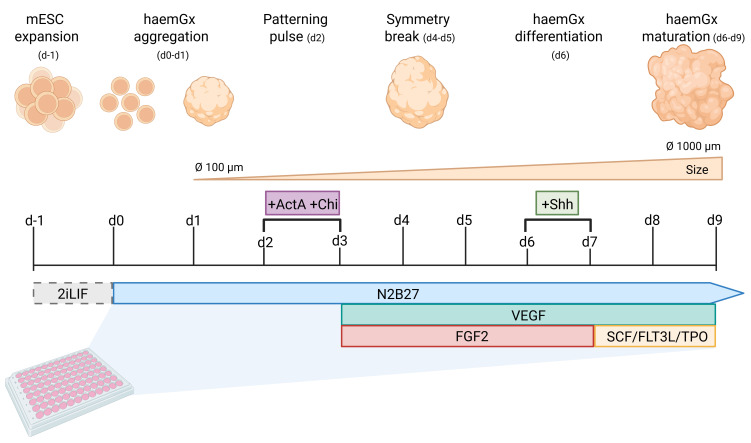


**Overview of the mouse hemogenic gastruloid (haemGx) model**

## Background

Understanding early embryonic development and hematopoietic specification remains a challenge in developmental biology and regenerative medicine and is central to the efficient generation of transplantable hematopoietic stem cells (HSC). Mouse embryonic stem cells (mESC) provide a powerful platform to model these processes in vitro due to their capacity for directed 2- and 3-dimensional differentiation into derivatives from the three germ layers, including blood. Over the past decades, 3D culture systems such as embryoid bodies (EBs) and, more recently, gastruloids, have been developed to recapitulate aspects of post-implantation development, including symmetry breaking, axial organization, and lineage specification [1].

While these systems have significantly advanced the field, they present important limitations. Conventional EBs lack spatial organization and reproducibility, which limits their ability to recapitulate developmental niche topology. Gastruloid models are capable of symmetry breaking and faithful body axis patterning, but they present biases in anterior–posterior lineage outputs, depending on culture conditions, and earlier versions of the protocol had infrequent and incomplete recapitulation of blood development [2].

The haemGx system described here addresses these challenges by enabling reproducible generation of hemato-endothelial structures and blood progenitor formation in gastruloids with a modified protocol to enable more balanced generation of anterior and posterior derivatives [3]. In terms of hematopoietic specification, the key advantage of this protocol is its ability to mirror extra-embryonic (yolk sac, YS-like) and intra-embryonic (aorta-gonad-mesonephros, AGM-like) waves of blood formation, approximating developmental trajectories observed in vivo. Critically, the protocol is compatible with multiple mESC lines, increasing its applicability across different experimental contexts. The use of 96-well plates allows scalability for high-throughput downstream assays, namely systematic screening of genetic and chemical perturbations, or further refinement of culture conditions to optimize specific combinatorial lineage outputs. Beyond modeling early hematopoiesis, this protocol can be applied to study lineage specification, gene function, and the effects of signaling perturbations in a controlled 3D environment. It also provides a useful platform for investigating mechanisms of developmental disorders affecting blood formation.

## Materials and reagents

**Biological materials**

1. ES-E14TG2a (E14) *Vegfr2^+/egfp^* mouse embryonic stem cells (mESC) [4]; herein, Flk1-GFP mESC line

**Reagents**

1. Glasgow’s MEM (GMEM) (Gibco, catalog number: 11710035)

2. Fetal bovine serum (FBS), embryonic stem cells tested (Biosera, catalog number: FB-1001S)

3. GlutaMAX supplement (100×) (Gibco, catalog number: 35050061)

4. MEM non-essential amino acids solution (100×) (Gibco, catalog number: 11140050)

5. Sodium pyruvate (100 mM) (Gibco, catalog number: 11360039)

6. 2-Mercaptoethanol (50 mM), cell culture grade (Gibco, catalog number: 31350010)

7. Neurobasal medium (Gibco, catalog number: 21103049)

8. DMEM/F-12, GlutaMAX supplement (Gibco, catalog number: 31331028)

9. N-2 supplement (100×) (Gibco, catalog number: 17502-048)

10. B-27 supplement (50×), serum-free (Gibco, catalog number: 17504-044)

11. Trypsin-EDTA (0.25%), phenol red (Gibco, catalog number: 25200056)

12. TrypLE Select Enzyme (1×), no phenol red (Gibco, catalog number: 12563011)

13. Bovine serum albumin (BSA), cell culture grade (ThermoFisher, catalog number: 10829410)

14. Dimethyl sulfoxide (DMSO), cell culture grade (Fisher BioReagents, catalog number: 10103483)

15. PBS with calcium and magnesium +/+ (Gibco, catalog number: 14040117)

16. PBS without calcium and magnesium -/- (Gibco, catalog number: 14190144)

17. Gelatin from porcine skin, Type A, powder, cell culture grade (Merck, catalog number: G1890)

18. LIF recombinant mouse protein, embryonic stem cell qualified (100 μg) (Gibco, catalog number: A35935)

19. StemMacs PD0325901 (Miltenyi Biotec, catalog number: 130-106-5411)

20. Recombinant human Activin A Plus protein (ACTA2) (100 μg) (Qkine, catalog number: Qk005-0100)

21. Chiron (CHIR99021) (10 mg) (Biogems, catalog number: 2520691)

22. Murine VEGF-165 (Peprotech, catalog number: 450-32-5UG)

23. Murine FGF-basic (Peprotech, catalog number: 450-33-10UG)

24. Murine Sonic Hedgehog protein (Shh) (Peprotech, catalog number: 315-22-20UG)

25. Murine SCF (Peprotech, catalog number: 350-03-20UG)

26. Murine FTL3-ligand (Flt3L) (Peprotech, catalog number: 250-31-50UG)

27. Murine TPO (Peprotech, catalog number: 315-14 -10UG)

28. Isopropanol (Thermo Fisher Scientific, catalog number: 149320025)

29. Trypan blue (Gibco, catalog number: 15250061)

**Solutions**

1. Reconstitution of cytokines (see Recipes)

2. Gelatin (10× and 1×) (see Recipes)

3. ESLIF medium (50 mL) (see Recipes)

4. N2B27 medium for d0 and d1 (see Recipes)

5. 2iLIF medium (10 mL) (see Recipes)

6. Freezing medium for mESC (see Recipes)

7. HaemGx differentiation medium d2 (see Recipes)

8. HaemGx differentiation medium d3–d5 (see Recipes)

9. HaemGx differentiation medium d6 (see Recipes)

10. HaemGx differentiation medium d7–d8 (see Recipes)

**Recipes**

**1. Reconstitution of cytokines**

Lyophilized cytokines and growth factors must be reconstituted and diluted in working aliquots to avoid thaw-freeze cycles, which can compromise activity. The first reconstitution is in sterile distilled water at 10× of the desired working solution; the 10× stock is further diluted in PBS + 0.1% BSA for working aliquots to be stored at -20 °C. Once thawed, individual aliquots can be kept in the fridge (2–8 °C) for up to 4 weeks.

**Critical:** Cytokine activity can vary by supplier and by lot. It is recommended to refer to the certificate of analysis (CoA) for information about the range of biological activity (e.g., ED50, U/mg).

*Note: The authors suggest preparing working aliquots of cytokines/growth factors containing the total volume required for one culture plate over the entire duration of the experiment, following the specification found in the “Quantity or volume” column of the tables. For example, VEGF is required for 6 days of the protocol at a volume of 10 μL per plate per day; therefore, prepare working aliquots of 60 μL, with an additional 2–3 μL to compensate for pipetting losses. Thaw a single aliquot at the start of the experiment and store it at 2–8 °C for subsequent use throughout the culturing period (9 days).*

**2.1 Gelatin (1%)**

**Table:** 

Reagent	Final concentration	Quantity or volume
Gelatin (powder)	1%	1 g
Double-distilled water	99%	100 mL
Total		100 mL

Prepare a 1% (w/v) gelatin stock solution by dissolving gelatin in double-distilled water. Heat gently (e.g., at 37–60 °C) to fully dissolve. Sterilize by filtration or autoclaving and store at room temperature (RT).

**2.2 Gelatin (0.1%)**

**Table:** 

Reagent	Final concentration	Quantity or volume
Gelatin (1%)	10%	10 mL
PBS	90%	90 mL
Total		100 mL

To prepare the working concentration (0.1%) of gelatin, dilute 10 mL of the stock concentration (1%) with 90 mL of PBS. Prepare a 10 mL/aliquot and store at 2–8 °C. Both 1% and 0.1% aliquots can be stored at 2–8 °C for ≤6 months.

**3. ESLIF medium (50 mL)**

**Table:** 

Reagent	Final concentration	Quantity or volume
GMEM	1×	43.35 mL
FBS	10%	5 mL
GlutaMAX supplement (100×)	1:100	500 μL
MEM non-essential amino acids solution (100×)	1:100	500 μL
Sodium pyruvate (100 mM)	1 mM	500 μL
2-Mercaptoethanol (50 mM)	0.1 mM	100 μL
LIF recombinant mouse protein (25 μg/mL)	25 ng/mL	50 μL
Total		50 mL

Protect the medium from light, as it is light-sensitive. Add all the reagents to a 50 mL conical tube and mix by gently inverting the tube 2–3 times. Warm up the medium in a 37 °C water bath before use. The medium can be prepared and stored at 2–8 °C for up to two weeks. For long-term storage of the medium, it is recommended to prepare the ESLIF medium without LIF, as its biological activity degrades over time when in solution; add LIF supplement before starting to culture cells.

**4. N2B27 medium for d0 and d1**

**Table:** 

Reagent	Final concentration	Quantity or volume
DMEM/F-12, GlutaMAX supplement	1×	24.35 mL
Neurobasal medium	1×	24.35 mL
GlutaMAX supplement (100×)	1:100	500 μL
B-27 supplement (50×)	1:50	500 μL
N-2 supplement (100×)	1:200	250 μL
2-Mercaptoethanol (50 mM)	50 μM	50 μL
Total		50 mL

Protect the medium from light, as it is light-sensitive. Add all the reagents to a 50 mL conical tube and mix by gently inverting the tube 2–3 times. Pre-equilibrate the medium in a 37 °C water bath before use. The medium can be prepared and stored at 2–8 °C for up to two weeks.

*Note: While it is possible to use commercially available N2B27 medium, we have observed large batch-to-batch variation requiring batch testing. We recommend the use of home-made N2B27 as shown in the recipe above.*

**5. 2iLIF medium (10 mL)**

**Table:** 

Reagent	Final concentration	Quantity or volume
N2B27	1×	10 mL
PD0325901 (10 mM)	1 μM	1 μL
Chiron (chir99021) (10 mM)	3 μM	3 μL
LIF		10 μL
Total		~10 mL

Protect components from light and prepare fresh aliquots immediately before use. Add all components to the basal medium (N2B27) and mix gently by inversion. Pre-equilibrate the medium in a 37 °C water bath before use.

2iLIF medium was originally described to maintain mESC in a state of naïve, i.e., unprimed, pluripotency [5]. It also enables ESC derivation from multiple species. Here, it is used to reset mESC to a more homogeneous pluripotent state (see Figure 1).

**Figure 1.**
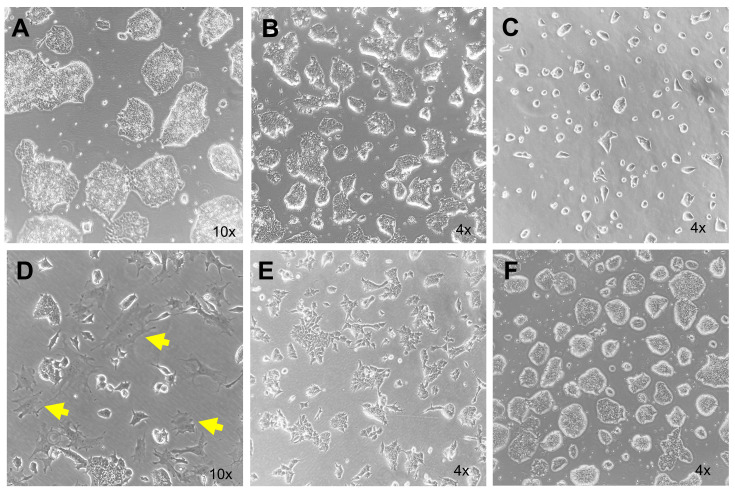
Morphological assessment of mouse embryonic stem cells (mESC) states prior to haemGx formation. (A) Optimal mESC culture morphology in ESLIF medium. Well-defined, round, and uniformly phase-bright colonies, with minimal attachment between colonies (confluency 50%–60%), indicate a robust pluripotent state. (B) Optimal mESC culture confluency at 50%–60% and pluripotent morphology in ESLIF medium; optimal for gastruloid assembly. (C) Low confluency mESC culture with appropriate pluripotent morphology; additional culturing is recommended to reach 50%–60% confluency. (D) mESC initiating differentiation. Colonies appear irregular with poorly defined, spiky edges. Individual cells within the culture exhibit a flattened morphology and reduced light refractility; differentiating cells are indicated by arrows. Under these conditions, haemGx assembly is not recommended. (E) mESC primed for differentiation. Colonies begin to lose compactness, but cells can still be recovered and reverted to a more pluripotent state. (F) mESC after one passage in 2iLIF medium. Colonies regain a rounded morphology, with cells appearing compact and phase-bright, displaying the characteristic halo-like refractive appearance.

**6. Freezing medium for mESC**

**Table:** 

Reagent	Final concentration	Quantity or volume
ESLIF medium	50%	500 μL
FBS	40%	400 μL
DMSO	10%	100 μL
Total		1 mL

Prepare the freezing medium fresh before use.

**7. HaemGx differentiation medium d2**

**Table:** 

Reagent	Final concentration	Quantity or volume
N2B27	1×	10 mL
Recombinant human Activin A Plus protein (ACTA2) (100 μg/mL)	100 ng/mL	10 μL
Chiron (chir99021) (10 mM)	3 μM	3 μL
Total		~10 mL

For day 2 pulse, supplement fresh N2B27 medium with Activin A and CHIR99021 immediately before use.

Activin A (a TGF-β pathway agonist) and CHIR99021 (a Wnt/β-catenin pathway activator via GSK3 inhibition) are used in combination to induce mesoderm specification and promote balanced anterior–posterior patterning [6]. Because of this balanced patterning of anterior–posterior mesoderm, it should be noted that hemogenic gastruloids do not elongate to the same extent as conventional gastruloids that are heavily posteriorized [7]. This coordinated signaling input is critical to initiate hematopoietic differentiation and to ensure proper spatiotemporal development of haemGx, putatively enabling capture of YS-like and AGM-like blood formation.

**8. HaemGx differentiation medium d3–d5**

**Table:** 

Reagent	Final concentration	Quantity or volume
N2B27	1×	10 mL
Murine VEGF-165 (5 μg/mL)	5 ng/mL	10 μL
Murine FGF-basic (10 μg/mL)	5 ng/mL	5 μL
Total		~10 mL

On days 3–5, supplement fresh N2B27 medium with VEGF and FGF2 immediately before use.

VEGF promotes endothelial and hematopoietic lineage specification, while FGF2 supports cell survival, proliferation, and mesoderm differentiation progression [8]. Together, these signals enhance the emergence and expansion of hematopoietic progenitors within the haemGx system.

**9. HaemGx differentiation medium d6**

**Table:** 

Reagent	Final concentration	Quantity or volume
N2B27	1×	10 mL
Murine VEGF-165 (5 μg/mL)	5 ng/mL	10 μL
Murine FGF-basic (10 μg/mL)	5 ng/mL	5 μL
Murine Sonic Hedgehog protein (Shh) (20 μg/mL)	20 ng/mL	10 μL
Total		~10 mL

At day 6, supplement fresh N2B27 medium with VEGF, FGF2, and Shh immediately before use.

Shh signaling is critical to the patterning of the dorsal aorta to enable hematopoietic specification from hemogenic endothelium [9]. Accordingly, it promotes late-stage progenitor output in haemGx [3]

**10. HaemGx differentiation medium d7–d8**

**Table:** 

Reagent	Final concentration	Quantity or volume
N2B27	1×	10 mL
Murine VEGF-165 (5 μg/mL)	5 ng/mL	10 μL
Murine SCF (20 μg/mL)	20 ng/mL	10 μL
Murine Flt3-ligand (50 μg/mL)	100 ng/mL	20 μL
Murine TPO (10 μg/mL)	20 ng/mL	20 μL
Total		~10 mL

On days 7 and 8, supplement fresh N2B27 medium with VEGF and hematopoietic cytokines (SCF, Flt3L, TPO) immediately before use. These cytokines support the expansion of HSC and multipotent progenitors in vitro [10]. They enhance CD45^+^ cell output in haemGx.

**Laboratory supplies**

1. Cell culture plate, 6-well, surface: standard, flat base (Sarstedt, catalog number: 83.3920.005)

2. Non-binding 96-well U-bottom microplates (Greiner, catalog number: 650901)

3. 15 mL conical centrifuge tubes (Sarstedt, catalog number: 62.554.502)

4. 50 mL conical centrifuge tubes (Sarstedt, catalog number: 62.547.254)

5. 1.5 mL microcentrifuge tubes (Sarstedt, catalog number: 72.690.001)

6. CryoPure tubes (Sarstedt, catalog number: 72.379)

7. Sterile reagent reservoirs (Thermo Fisher Scientific, catalog number: 10618012)

8. 5 mL serological pipettes (Sarstedt, catalog number: 86.1253.001)

9. 10 mL serological pipettes (Sarstedt, catalog number: 86.1254.001)

10. 25 mL serological pipettes (Sarstedt, catalog number: 86.1685.001)

11. 20 μL pipette tips (Sarstedt, catalog number: 70.760.213)

12. 200 μL pipette tips (Sarstedt, catalog number: 70.3030.305)

13. 1,000 µL pipette tips (Sarstedt, catalog number: 70.762.211)

14. Wide-bore pipette tips (Thermo Fisher Scientific, catalog number: 2069G)

## Equipment

1. Biosafety cabinet (Scientific Laboratory Supplies, model: Faster SafeFAST Premium 218, catalog number: CAB1576)

2. CO_2_ incubator (Binder, catalog number: BIN-CB56-115)

3. Inverted light microscope (Motic, model: AE2000 binocular, catalog number: ATT4.1)

4. Benchtop fluorescence microscope with fluorescent channel (optional; required when using fluorescent reporter cell lines) (Leica, model: Mateo FL)

5. Bench-top centrifuge with 15/50 mL-conical tube holders (Thermo Fisher Scientific, model: Sorvall ST 8)

6. Microcentrifuge for 1.5 mL microcentrifuge tubes (Eppendorf, model: EP5420000164)

7. Water bath (Grant Instruments, model: JB Nova Digital)

8. Mechanical pipette (Fisher Scientific, catalog number: FB14955202)

9. Single-channel pipettes, P200 and P1000 (Starlab, ErgoOne series, catalog numbers: S7110-0200, S7110-1000)

10. Multi-channel pipette (Starlab, model: ErgoOne multichannel, catalog number: S7110-0208)

11. Hemocytometer (Neubauer chamber) (Thermo Fisher Scientific, catalog number: 02-671-10)

12. Controlled-rate freezing container (Mr. Frosty) (Thermo Fisher Scientific, catalog number: 5100-0001)

## Procedure

**A. Thawing and seeding mESCs**

**Critical:** When thawing mESC, allow at least one week of recovery, passaging, and expansion in 6-well plates before plating for experiments.

*Note: Authors recommend passaging cells 1–2 times every 3–4 days before plating for experiments. Passaging enhances viability and culture purity.*

1. Prepare a gelatin-coated 6-well plate by adding 1.5 mL of 0.1% gelatin (Recipe 2.2) per well. Incubate at room temperature (RT) for at least 15 min before seeding the cells.

*Note: Larger culture vessels, such as T25 tissue culture flasks with vent caps, can be used by scaling up reagents to match the surface area.*

2. After the incubation period, gently remove and discard the gelatin from the wells using a pipette.

**Critical:** Remove gelatin immediately before seeding to prevent the coated surface from drying out.

3. Prepare 15 mL conical tubes with 9 mL of prewarmed ESLIF medium (Recipe 3).

4. Remove the cryovial from liquid N_2_ storage and thaw for approximately 1 min in a 37 °C water bath until a small pea-sized frozen clump (around 0.5 cm) is left.

5. Collect the cell suspension from the cryovial and gently transfer to the 15 mL conical tube with the prewarmed ESLIF medium using a P1000 pipette dropwise.

6. Centrifuge at 300× *g* for 5 min at RT.

7. Carefully remove the supernatant and add 2 mL/well of prewarmed ESLIF. Carefully pipette up and down approximately 10 times to homogeneously disperse the cell pellet.

8. Seed the mESC at a density of 2 × 10^5^ to 5 × 10^5^ cells per well to maintain pluripotency. Use 2 mL of ESLIF medium per well.

**Critical:** Ensure that cells are free from clumps and resuspended to single cells in suspension by gently pipetting up and down 10 times using a P1000 pipette.

*Note: In the authors’ experience, 3* × *10^5^ is the optimal seeding density to keep the cells in culture for 2 days prior to gastruloid generation. The colony size shown in Figure 1C can serve as a reference for the day after reaching confluency.*

9. Gently rock the plate to ensure even distribution of the cells. Check cell density under an inverted light microscope.

10. Incubate at 37 °C with 5% CO_2_ for 24 h to allow cell attachment.

**B. mESC culture maintenance**

1. To maintain mESC cultures, replace the medium daily with fresh, prewarmed ESLIF medium (Recipe 3).

2. Gently remove and discard all medium from each well using a pipette.

3. Add 2 mL of fresh ESLIF medium to the well. Avoid disturbing attached cells by pipetting slowly onto the walls of the vessel.

4. Monitor cell morphology and confluence daily. mESC should be passaged every 2–3 days, before colonies reach full confluence and begin to merge into a continuous cell layer.

**Critical:** Pluripotent colonies appear compact with well-defined, rounded edges (Figure 1A). Optimal confluency of 60%–70% should be maintained (Figure 1B; underconfluent cultures appear as Figure 1C); differentiated cells exhibit a flattened, irregular, or “spiky” morphology (Figure 1D). If signs of differentiation are observed (>10%–20% of differentiated cells observed) or if cells appear primed to differentiate (Figure 1D–E), cultures can be treated with 2iLIF medium (Recipes 4–5) for 24–48 h (with daily medium change) to maintain or restore the pluripotent state (Figure 1F).

**C. Passaging mESC**

1. Prewarm ESLIF medium, trypsin-EDTA, and PBS+/+ at 37 °C prior to starting.

2. Prepare a gelatin-coated 6-well plate by adding 1.5 mL of 0.1% gelatin per well. Incubate at RT for at least 15 min before seeding the cells.

3. When cells are ready to be split (at >60% confluency), remove the existing ESLIF medium from the well and discard it.

*Note: To avoid the introduction of bubbles during the splitting process, the use of pipettes (P1000) is preferred over a pipettor.*

4. Wash the cells by adding 2 mL of PBS+/+ to the well and gently rocking the plate. To avoid forced cell detachment, add PBS along the wall of the well rather than directly onto the cell layer.

5. Add 100 μL of trypsin-EDTA to cover the well, gently rock the plate to distribute the solution, and incubate at 37 °C with 5% CO_2_ for 3–5 min, until all cells detach as single cells or small clumps.

6. After incubation, quench the trypsin-EDTA by adding 3 mL of ESLIF medium (the medium must contain serum to inactivate the enzymatic activity of the trypsin) to the well. Pipette up and down using a P1000 pipette until the cells are fully resuspended into a single-cell suspension with no remaining visible clumps.

7. Transfer the cell suspension to a conical 15 mL tube and add ESLIF medium to reach a volume of 5 mL.

8. Centrifuge at 400× *g* for 5 min.

*Note: To ensure the cells are free of gelatin, an optional washing step with PBS+/+ after centrifuging in step C8 is recommended by decanting the medium without disturbing the pellet and resuspending in 5 mL of PBS+/+. Centrifuge at 400×* g *for 5 min to pellet.*

9. Carefully aspirate the supernatant and resuspend the cell pellet in 1 mL of ESLIF medium using a P1000 pipette.

10. Count the cells using a viability dye such as Trypan blue using a hemocytometer.

*Note: A typical splitting ratio for mESC ranges from 1:4 to 1:8 from 60% confluency. Viability above 90% indicates a healthy culture.*

11. Plate cells by adding the appropriate volume of cell suspension for a seeding density of 2 × 10^5^ to 5 × 10^5^/well. Adjust with ESLIF medium to a final volume of 2 mL per well.

12. Gently rock the plate and incubate at 37 °C with 5% CO_2_ overnight.

13. Continue culture maintenance as described in **section A** with daily medium changes.

**D. mESC 2iLIF treatment**

**Critical:** Treat with 2iLIF in case of differentiated morphology (see Figure 1 for possible morphologies). 2iLIF treatment should not exceed 24–48 h. A transient 2i treatment is sufficient to recover pluripotency and to counteract early signs of differentiation.

*Note: In the authors’ experience, some mESC lines can respond rapidly to 2i conditions, and in most cases, simply replacing ESLIF culture medium with 2iLIF is sufficient to achieve a homogeneous reset of pluripotent colony morphology (Figure 1F). This is why the medium replacement approach is suggested as the first trial. However, some cell lines require a more robust approach of passaging the cells and seeding them directly into 2iLIF at the time of splitting. This approach promotes a more homogeneous response and improves culture stability. Familiarization with the mESC lines and respective cultivars is critical to decide on pluripotent morphology and the need and mode of 2i-LIF treatment. The authors recommend performing a pre- and post-treatment comparison (e.g., via imaging) of colony morphology to evaluate the response, using Figure 1F as a reference for the expected pluripotent state once reset is achieved.*

1. Follow **section C** for mESC passaging.

2. After counting the cells, add the appropriate volume to a 15 mL conical tube containing 1 mL of ESLIF medium.

3. Centrifuge at 400× *g* for 5 min.

4. Carefully discard the supernatant, resuspend the cell pellet in 2 mL of 2iLIF medium, and seed the cells in a gelatin pre-coated 6-well plate.

**Critical:** Seed a higher number of cells than the recommended density range of 2 × 10^5^ to 5 × 10^5^/well. Adjust empirically from 5 × 10^5^ to 1 × 10^6^. Differentiated cells are selectively lost under 2iLIF conditions, which can result in a significant reduction in viable cell numbers after treatment.

**E. Freezing mESC**

1. Follow **section C** to detach cells from vessels.

2. Prepare freezing medium according to Recipe 6. Store the freezing medium at 4 °C (on ice or in the fridge) to reduce DMSO toxicity.

3. Centrifuge cells at 300× *g* for 5 min at RT.

4. Carefully aspirate the supernatant.

5. Resuspend the cell pellet in pre-chilled freezing medium at a concentration of 1–2 × 10^6^ cells per mL by gently flicking the tube.

6. Dispense 1 mL of cell suspension into labeled cryovials.

7. Place cryovials into a controlled-rate freezing container filled with isopropanol (Mr. Frosty) and transfer to a -80 °C freezer. This ensures a cooling rate of approximately -1 °C per minute.

8. Transfer cryovials to liquid nitrogen for long-term storage the following day.

**F. Generation and differentiation of haemGx**

*Note: The haemGx differentiation protocol spans 10 days from assembly (d0) to endpoint (d9) (Figure 2). Individual haemGx are assembled in a 96-well plate. A day-to-day summary of the protocol is reported in Table 1.*

**Critical:** mESC culture conditions must be monitored to ensure appropriate health and differentiation status before assembling haemGx. Confluency must not exceed 60%–70% on the day of assembly; inspect cultures visually under an inverted microscope for appropriate pluripotent morphology and treat cultures in 2iLIF if necessary (see Figure 1).

**Table 1.** haemGx protocol overview

Step	Component	Initial stock concentration	Final concentration	Volume to remove per well (µL)	Volume to add per well (µL)	Total (100 wells)
Day 0 (Seeding)	Cells in N2B27	—	400 cells/well	—	40	4 mL
Day 1 (Aggregation)	—	—	—	—	—	—
Day 2 (Pulse)	N2B27	—	—			15 mL
	Activin A	100 μg/mL	100 ng/mL	—	150	15 μL
	CHIR99021	10 mM	3 μM			3 µL
Day 3 (Stimulation)	N2B27	—	—			15mL
	mouse VEGF-165	5 μg/mL	5 ng/mL	150	150	15 μL
	FGF2	10 μg/mL	5 ng/mL			7.5 μL
Days 4–5 (Maintenance)	N2B27	—	—			10 mL
	Mouse VEGF-165	5 μg/mL	5 ng/mL	150 d4 100 d5	100	10 µL
	FGF2	10 μg/mL	5 ng/mL			5 μL
Day 6 (Signaling)	N2B27	—	—			10 mL
	mouse VEGF-165 mouse	5 μg/mL	5 ng/mL	100	100	10 µL
	FGF2	10 μg/mL	5 ng/mL			5 μL
	Mouse SHH	20 μg/mL	20 ng/mL			10 mL
Days 7–9 (Maturation)	N2B27	—	—			10 mL
	VEGF-165	5 μg/mL	5 ng/mL			10 µL
	SCF		100 ng/mL	100	100	10 µL
	Flt3L		100 ng/mL			10 µL
	TPO		20 ng/mL			10 µL

1. Prewarm ESLIF medium, N2B27 medium (Recipe 4), and Trypsin-EDTA in a 37 °C water bath.

2. Remove and discard the ESLIF medium from the mESC culture.

3. Wash the cells by adding 2 mL of PBS+/+ to the well and gently rock the plate. To avoid detaching the cells, add the PBS along the wall of the well rather than directly onto the cell layer.

4. Add 100 μL of trypsin-EDTA to cover the well, gently rock the plate to distribute the solution, and incubate at 37 °C with 5% CO_2_ for 2–3 min, until all cells detach.

5. After incubation, quench trypsin by adding 3 mL of ESLIF to the well. Pipette up and down using a P1000 pipette.

*Note: Pipette cells until they are fully resuspended and no visible clumps remain.*

6. Transfer the cell suspension to a conical 15 mL tube and top up with ESLIF to reach a volume of 5 mL.

7. Centrifuge at 400× *g* for 5min.

8. Carefully aspirate the supernatant and resuspend the cell pellet in 5 mL of PBS +/+.

9. Centrifuge at 400× *g* for 5min.

10. Repeat steps F8–9.

**Critical:** Washing twice with PBS is essential to ensure the removal of traces of FBS from the medium, as N2B27 is serum-free.

11. Carefully aspirate the supernatant and resuspend in 1 mL of prewarmed N2B27 medium.

12. Count the cells and determine viability using a viability dye (Trypan blue) using a hemocytometer.

**Critical:** Viability must exceed 90%.

13. To generate haemGx, seed 400 mESC per well in 40 μL of N2B27 medium, using a non-binding, ultra-low adherence, U-bottom, 96-well plate.

**Critical:** The use of an ultra-low adherence, non-binding, U-bottom microplate is essential. During the first 24 h of incubation, the cells will self-organize; the appropriate plate enables proper aggregation and formation of a spherical 3D structure, which is maintained in suspension throughout the protocol (Figure 2C).

14. Prepare the cell suspension required for seeding.

*Note: For a full 96-well plate at 400 cells per well, a total of 38,400 cells is required. Include additional volume to reach the equivalent of 100 wells to account for pipetting errors, resulting in 40,000 cells in total.*

15. Bring the suspension to the final volume required to fill the microplate.

*Note: For a full 96-well plate at 40 μL per well, a total of 3.84 mL is required. Include additional volume to reach the equivalent of 100 wells to account for pipetting errors, resulting in 4 mL in total.*

16. Mix thoroughly by pipetting up and down approximately 10 times using a P200 pipette to ensure a homogeneous, single-cell suspension.

17. Transfer the suspension to a sterile reservoir and seed 40 μL per well using a multichannel pipette.

18. Incubate the cells in a humidified incubator at 37 °C and 5% CO_2_ for 48 h (2 days) to allow haemGx aggregation (day 0, d0).

*Note: After two days, examine haemGx under a light microscope. Properly formed haemGx appear as round, spherical structures with defined borders (Figure 2B). HaemGx will grow in size and will acquire an ovoid shape, followed by irregular internal structures over the course of the protocol; morphology can be monitored using an inverted light microscope (Figure 2C).*

19. On d2, pulse the haemGx with Activin A and CHIR99021. Prepare fresh N2B27 medium supplemented with 100 ng/mL Activin A and 3 μM CHIR99021 according to Recipe 7. Add 150 µL of medium per well without removing the existing medium.

**Critical:** Because the haemGx are in suspension, not embedded in a synthetic matrix, medium addition and removal must be performed slowly (approximately 3 s per well) to avoid disturbing the 3D structure.

*Notes:*

*1. Keep the tip of the P200 pipette tilted at ~30° so that it does not touch the haemGx at the bottom of the well (please refer to [11] for detailed pipette positioning).*

*2. For a full 96-well plate at 150 μL per well, a total of 14.4 mL is required. Include additional volume to reach the equivalent of 100 wells to account for pipetting error, resulting in 15 mL in total. For 15 mL of N2B27 supplemented medium, 15 μL of Activin A and 4.5 μL of CHIR99021 are required.*

20. Incubate for 24 h in a humidified incubator at 37 °C and 5% CO_2_.

21. On d3 of the protocol, remove 150 µL of N2B27 medium from the well using a multichannel pipette.

22. Add 150 μL per well of fresh prewarmed N2B27 medium supplemented with 5 ng/mL of mouse VEGF-165 and 5 ng/mL mouse FGF2 (Recipe 8).

**Critical**: From d0 to d3, all steps must follow a strict 24-h schedule to ensure reproducible haemGx development, with symmetry breaking and establishment of the body plan. From d4 onward, medium changes can be performed with a ±2 h window from the previous day without affecting the experiment.

23. On d4, remove 150 μL of N2B27 medium from the well using a multichannel pipette.

*Note: At d4, haemGx become ovoid in shape (Figure 2C), and symmetry breaking occurs. Using a Flk1-GFP reporter, this is visualized by polarized patterning of the Flk1 signal, signifying induction of hemato-endothelial lateral plate mesoderm (see Validation of protocol).*

24. Add 100 μL per well of fresh prewarmed N2B27 medium supplemented with 5 ng/mL of mouse VEGF-165 and 5 ng/mL mouse FGF2 (Recipe 8).

25. On d5, remove 100 µL of N2B27 medium from the well using a multichannel pipette.

26. Add 100 μL per well of fresh prewarmed N2B27 medium supplemented with 5 ng/mL of mouse VEGF-165 and 5 ng/mL of mouse FGF2 (Recipe 8).

27. On d6, remove 100 μL of N2B27 medium from the well using a multichannel pipette.

*Note: At d6, check the expression of CD41 by flow cytometry to ensure correct emergence of early hematopoietic progenitors, representing YS-like hematopoiesis (see Validation of protocol).*

28. Add 100 μL per well of fresh prewarmed N2B27 medium supplemented with 5 ng/mL of mouse VEGF-165, 5 ng/mL mouse FGF2, and 20 ng/mL of mouse Sonic Hedgehog protein (Shh) (Recipe 9).

29. On d7, remove 100 μL of N2B27 medium from the well using a multichannel pipette.

30. Add 100 μL per well of fresh prewarmed N2B27 medium supplemented with 5 ng/mL of mouse VEGF-165, 100 ng/mL of mouse SCF, 100 ng/mL of mouse Flt3-ligand, and 20 ng/mL of mouse TPO (Recipe 10).

31. On d8, repeat steps F29–30.

32. On d9, haemGx can be dissociated and/or collected for downstream experiments.

*Note: At d8–9, check the expression of CD45 by flow cytometry to ensure correct emergence of AGM-like hematopoietic output (see Validation of protocol).*

**Figure 2.**
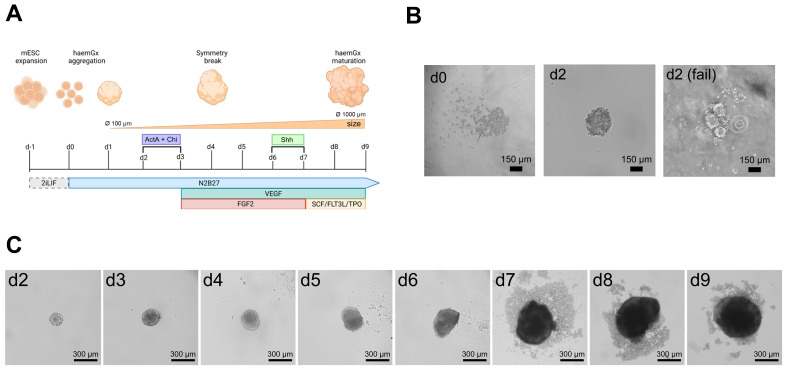
HaemGx protocol overview. (A) Schematic representation of the hemogenic differentiation from mouse embryonic stem cells (mESC) to haemGx. HaemGx are assembled in 96-well plates with a daily N2B27 medium regimen of supplementations to promote hemato-endothelial and hematopoietic specification. (B) Representative image of mESC seeded at d0 for haemGx assembly (d0, left panel). At d2, an appropriately assembled haemGx with a defined spherical structure is shown in the middle panel, compared to a failed assembly with multiple aggregates lacking structural definition (right panel; refer to Table 2 for troubleshooting). Magnification, 10×. (C) Overview of haemGx morphology by timepoint. Between d7 and d9, a variable amount of shedding can be observed, which does not impact the successful hematopoietic output. Shed and unshed cells are collected together for downstream analyses.

**G. Dissociation of haemGx for downstream analyses**

*Note: HaemGx can be dissociated at any time point to investigate specific temporal windows.*

1. Prewarm ESLIF medium, PBS -/-, and TrypLE in a 37 °C water bath.

2. To collect haemGx, transfer individual haemGx from each well to a 1.5 mL microcentrifuge tube using a P1000 pipette. As a reference, one full plate (96 haemGx) can be pooled in one 1.5 mL tube.

**Critical:** Use a P1000 pipette tip when handling haemGx. The typical diameter of a haemGx does not exceed 1 mm, which is the opening of the P1000 tip, allowing safe transfer of all the cells.

*Note: To preserve the 3D structure (e.g., for microscopy imaging), the tip of the p1000 pipette can be cut with a sterile scissor, or wide-bore tips can be used to increase the opening and minimize the risk of damaging the haemGx.*

3. Wait for 2 min until the haemGx sediments to the bottom of the microcentrifuge tube (**Figure 3**).

4. Carefully discard the excess supernatant.

*Note: To promote sedimentation, the microcentrifuge tube can be centrifuged at 300*× *g for 3 min.*

5. Add 1 mL of PBS -/- to the microcentrifuge tube and gently flick the bottom of the tube to resuspend the haemGx.

6. Centrifuge at 400× *g* for 5 min.

7. Carefully remove and discard the supernatant.

8. Add 200 μL of TrypLE to the microcentrifuge tube and gently flick the tube.

*Note: 200 μL of TrypLE is sufficient to dissociate the haemGx content of half of a 96-well plate, corresponding to 48 haemGx. Scale volumes as needed.*

9. Incubate the sample at 37 °C in a humidified incubator with 5% CO_2 _for 5 min.

*Note: Alternatively, the incubation can be performed in a water bath at 37 °C for 5 min.*

10. After the incubation period, vigorously resuspend the haemGx with a P200 pipette to ensure separation of the cells and to dislodge any visible clumps.

11. Once the cell suspension is homogeneously dispersed, add 600 μL of ESLIF medium to quench the TrypLE.

12. Centrifuge at 400× *g* for 5 min to pellet.

13. Carefully remove and discard the supernatant.

14. Resuspend the cell pellet and prepare the cells for further analysis, according to the experimental plan.

*Note: The estimated yield for each haemGx is 30,000–50,000 cells at d9.*

**Figure 3.**
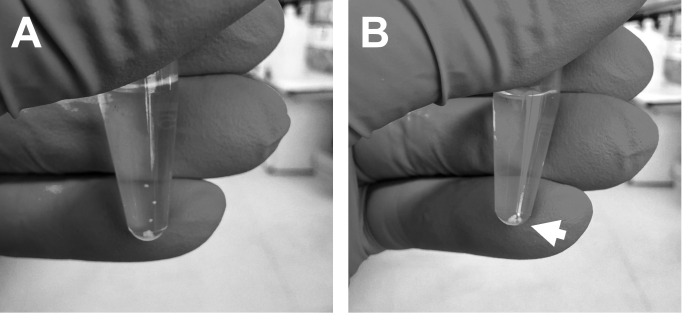
Collection of haemGx for disassembly. (A) Individual haemGx are transferred to microcentrifuge tubes using P1000 pipettes, maintaining their integrity. HaemGx will deposit at the bottom of the tube by gravity until (B) fully collected in 1–2 min.

## Data analysis

Downstream analyses of haemGx can include a range of molecular, biochemical, and imaging assays. Refer to [3] for examples of flow cytometry, single-cell and bulk RNA-sequencing, and colony-forming assays. Immunofluorescence staining protocols for gastruloids have been described elsewhere [11]. Refer to [12] for computational approaches combining haemGx and clinical transcriptomics data for cell-of-origin mapping for leukemias of embryonic origin.

## Validation of protocol

This protocol has been used and validated in the following research article:

- Ragusa et al. [3]. Dissecting infant leukemia developmental origins with a hemogenic gastruloid model. *eLife.*
  This protocol has been optimized for the stepwise recapitulation of key events in embryonic hematopoiesis by the following read-outs as successful achievement of the differentiation process:
  1. Symmetry breaking and endothelial patterning at d4, which can be visualized by the reporter Flk1-GFP by fluorescence microscopy (**Figure 4**).
  2. Partial elongation with acquisition of reproducible ovoid shape at d4–5 (**Figure 2C**). In the case of the *Flk1-GFP* reporter line, this time coincides with an extension of the GFP+ area at one pole of the haemGx.
  3. Emergence of CD41+ early hematopoietic progenitor populations at d6 (**Figure 5**).
  4. Emergence of CD45+ hematopoietic output between d8 and d9 (endpoint) (**Figure 5**).

Endothelial patterning requires the pulse of Activin A and CHIR99021 at d3, as demonstrated by the failure to express Flk1 when omitted, which results in failure to achieve hematopoietic output at endpoint (Supplementary Figure 1 from [3]). There is high well-to-well reproducibility in timing and polarization of the signal (**Figure 4**). The endothelial network has been validated by flow cytometry and immunofluorescence for co-expression with the endothelial marker CD31 (Figure 2 from [3]).

**Figure 4.**
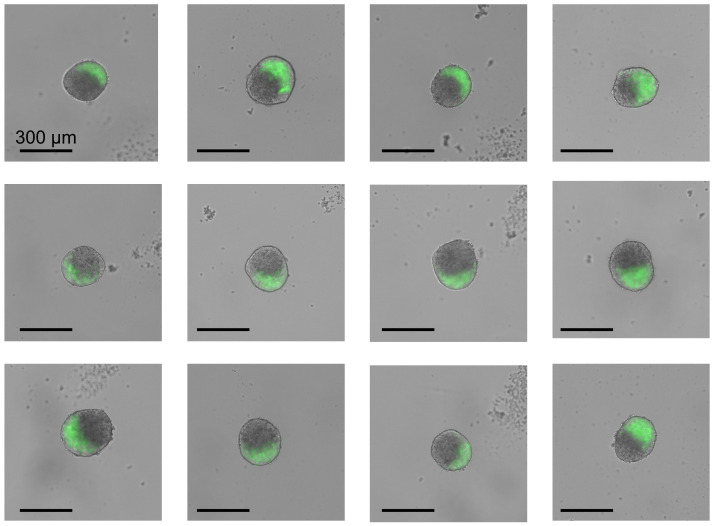
Polarization of Flk1-GFP signal in haemGx at day 4. Representative images of individual haemGx with correct patterning of hemato-endothelial lateral plate mesoderm by polarized signal of Flk1-GFP. Magnification, 4×.

The output of CD45^+^ at endpoint is detectable from d8 by flow cytometry at ranges from 0.5% to 4% (described in detail in [3]). The protocol has been optimized to increase the CD45^+^% by the addition of a cytokine cocktail of SCF, Flt3L, and TPO (Supplementary Figure 1 from [3]). The CD45^+^ hematopoietic output at endpoint has been characterized by multi-color flow cytometry and single-cell RNA-sequencing, confirming co-expression of c-Kit, Flk1, VE-cadherin, and CD34, which is consistent with definitive hematopoietic stem and progenitor cells (Figures 2–3 from [3]). The putative AGM-like nature of CD45^+^ cell production was supported by the lack of responsiveness to EZH2 inhibition; this was in contrast with the earlier CD41^+^ wave, which required EZH2 activity, suggestive of pre-definitive YS-like hematopoiesis (Supplementary Figure 2–2 from [3]). Comparison with published scRNA-seq data was compatible with the capture of two waves (Figure 4 from [3]). Functional validation at endpoint was performed by hematopoietic colony-forming assays showing the ability to form multi-lineage colonies (Figure 2E from [3]), as well as by in vivo implantation of haemGx via in situ maturation in adrenal glands producing low-level multi-lineage engraftment (Figure 4 from [3]). This protocol is reproducible using other mESC lines and amenable to the use of engineered reporter lines. The time-dependent expression of CD41 and CD45 has been validated using E14 and multiple reporter ESC lines in different genetic backgrounds (Supplementary Figure 1 from [3]).

**Figure 5.**
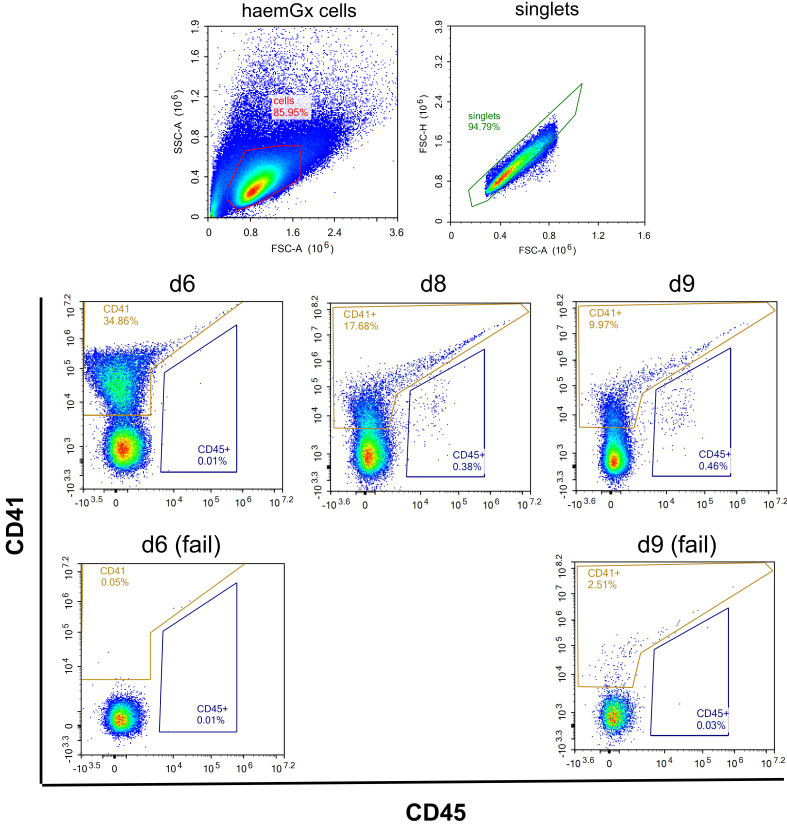
Temporal expression of CD41 and CD45 in haemGx. Representative flow cytometry plots showing the expression of CD41 and CD45 at day 6 (d6), d8, and d9. CD41 is expressed at d6 with no CD45^+^ output, indicating the correct differentiation profile of early hematopoietic progenitors. Between d8 and d9, CD41 expression decreases, and CD45^+^ hematopoietic progenitors arise. Absence of CD45^+^ at d9 indicates a failure in hematopoietic specification in haemGx.

## General notes and troubleshooting

**General notes**

1. All cytokines, growth factors, and sensitive reagents should be aliquoted upon receipt to avoid repeated freeze–thaw cycles. Many growth factors lose activity with repeated freezing and thawing; small aliquots help maintain protein stability and biological activity over time.

2. Maintain strict sterile technique throughout all procedures. Media used in these protocols do not contain penicillin/streptomycin; therefore, careful handling is essential to avoid contamination.

3. Because these protocols require consistent daily care, weekend handling can be minimized. mESCs can be passaged on Friday evening with a medium change on Sunday morning. For haemGx formation, experiments can also be initiated on Friday with the appropriate pulse applied on Sunday.

4. mESCs are prone to spontaneous differentiation during extended culture. Regular freezing of early-passage stocks is strongly recommended. Cells should typically be used below ~30 passages to ensure maintenance of pluripotency and reproducibility.

5. mESCs can be efficiently expanded in standard tissue culture vessels, including T25 flasks, without loss of quality when properly maintained.

6. Cells can be frozen in either 50% complete ESLIF medium (LIF optional) + 40% FBS (tested for ES cells) + 10% DMSO, or 90% ESLIF medium (LIF optional) + 10% DMSO. Serum improves post-thaw recovery but is not strictly essential.

7. When using full 96-well plates for haemGx formation, place plates in a tray with a lid inside the incubator to reduce evaporation. Alternatively, use only the 60 central wells and fill the outer wells with sterile PBS to maintain humidity and minimize edge effects.

8. For harvesting haemGx, ensure that pipette tips are at least ~1 mm in diameter. Alternatively, cut sterile tips using clean scissors. This prevents mechanical disruption and preserves gastruloid integrity during collection, maintaining their 3D structure.

9. Common points of failure and potential solutions are reported in Table 2. The main causes of failure of haemGx differentiation depend on mESC culture maintenance and/or the quality of media and its supplements.

**Troubleshooting**

**Table 2.** Troubleshooting of common fail points in haemGx

Problem	Possible cause	Potential solution
mESC viability is low (<70%)	Incorrect freezing/thawing of the vial	If possible, attempt to recover the culture by maintaining the cells in culture for one additional day and changing the medium as scheduled. Thaw another vial from the same freezing batch to assess whether the initial freezing was suboptimal.
mESC culture shows signs of spontaneous differentiation	Degradation of ESLIF components. Media stored past its shelf life. Extended cultures for more than 30 passages.	Prepare fresh ESLIF medium; ensure aliquots of inhibitors are not subjected to excessive freeze-thaw cycles.
mESCs fail to attach to the culture plate after seeding	Suboptimal gelatin coating (concentration or time). Incorrect incubator calibration (CO_2_ or temperature). DMSO toxicity during thawing.	Coat dishes with 0.1% gelatin in PBS for at least 15 min at RT. Ensure cells are immediately diluted in prewarmed medium after thawing to remove freezing medium.
HaemGx fail to aggregate into a single cluster	High density of starting cells; presence of air bubbles in the well. Low-quality U-bottom plates. mESC clumps in the starting suspension.	Use high-quality ultra-low-attachment U-bottom plates. Ensure that a single-cell suspension is achieved during the detachment of mESC. Centrifuge plates briefly, if necessary, to accelerate the movement of cells to the bottom of the wells.
HaemGx fail to polarize (e.g., no Flk1-GFP signal)	Incorrect concentration of the patterning pulse. Loss of activity of N2 and B27 supplements.	The Activin A activity is critical. Check the concentration and thaw a fresh aliquot. Start a new culture with freshly prepared N2 and B27 supplements.
HaemGx do not grow through timepoints	Incorrect medium preparation or expired components. Incorrect incubator calibration (CO_2_ or temperature).	Ensure that medium is prepared according to instructions and stored appropriately. Check the parameters of the incubator and, if needed, start a new culture in a new incubator. Ensure reduced incubator traffic to avoid frequent drops of CO_2_ and temperature. An incubator dedicated to gastruloid cultures is ideal.
HaemGx do not express correct differentiation markers	Incorrect medium preparation or expired components. Extended cultures for more than 30 passages or poor culture maintenance prior to aggregation.	Ensure that medium is prepared according to instructions and stored appropriately. Restart with a new batch of mESC and culture with care by examining morphology and confluency prior to aggregation. If differentiation checkpoints are not satisfied, restart cultures.
Significant loss of haemGx during medium exchange	High-velocity pipetting. Incorrect pipette angle. Uncalibrated equipment.	Use a calibrated pipette. Position the tip at a 30–45° angle against the well wall. Aspirate and dispense slowly to avoid disturbing the gastruloid at the center/bottom.
haemGx do not disassemble during processing	Insufficient enzymatic activity or mechanical force during dissociation.	Increase the volume of TrypLE Express and extend incubation time. Perform gentle mechanical dissociation by pipetting up and down with a P200 tip.
